# Supplementary figures and images for: A systematic in vitro study of the effect of normoglycaemic and hyperglycaemic conditions on the biochemical and cellular interactions of clinically-available wound dressings with different physicochemical properties
Source: PLoS One. 2025 Jan 24;20(1):e0317258. doi: 10.1371/journal.pone.0317258 (PMC11760615; doi:10.1371/journal.pone.0317258)

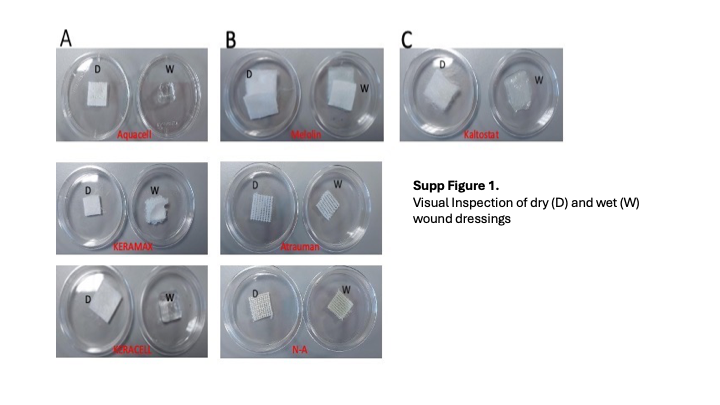

Supplement: S1 Fig — A: Dressings made of synthetic polymers, B: Carboxymethylcellulose- and cellulose-based dressings; C: Alginate-based dressing. (TIF) [file pone.0317258.s001.tif]
